# Macroscopic features of the kidneys of fetuses and newborns in preeclampsia: postmortem observational study

**Published:** 2018-02

**Authors:** Iryna Sorokina, Tetyana Ospanova, Mykhailo Myroshnychenko, Iryna Korneyko

**Affiliations:** 1 *Department of Pathological Anatomy, Kharkiv National Medical University, Kharkiv, Ukraine.*; 2 *Department of Propaedeutics of Internal Medicine No. 2 and Nursing, Kharkiv National Medical University, Kharkiv, Ukraine.*; 3 *Department of Foreign Languages, Kharkiv National Medical University, Kharkiv, Ukraine.*

**Keywords:** Fetus, Kidney, Macroscopy, Newborn, Preeclampsia

## Abstract

**Background:**

The state of the mother health is of great importance in the development of children renal pathology.

**Objective:**

To reveal macroscopic features of the fetuses and newborns kidneys of the mothers whose pregnancy was complicated by preeclampsia.

**Materials and Methods:**

The study involved the kidneys of 106 full-term fetuses and newborns of mothers with physiological pregnancy, complicated pregnancy by preeclampsia. During the autopsies, the shape, texture, surface, color were assessed in each kidney, and then its weight, length, width and thickness were measured.

**Results:**

The kidneys in fetuses and newborns from mothers with severe preeclampsia were characterized by a more pronounced lobulation. In fetuses and newborns from mothers with mild preeclampsia, the mass of the left kidney prevailed over the mass of the right kidney. The organometric kidney values were greater in newborns compared to fetuses in the cases of complicated by mild and moderate preeclampsia maternal pregnancy.

**Conclusion:**

Maternal moderate and severe preeclampsia delay renal growth in fetuses and newborns that manifest by a reduction of the kidneys organometric parameters.

## Introduction

Preeclampsia is a serious complication of pregnancy (1), which is characterized by hypertension, significant proteinuria, with or without edema. It is a multifactorial condition which forms an integral part of the continuum of hypertensive disorders of pregnancy. Preeclampsia complicates approximately 10% of pregnancies, but there are significant geographic variations in the incidence of this complication of pregnancy (2). The prevalence of preeclampsia was reported as 3.4% in the United States, 8.9% in Brazil, 3.3% in Australia, 12% in Bangladesh, 3.2% in India and 4.7% in Thailand. Preeclampsia is associated with higher rates of maternal, fetal and infant morbidity and mortality (3). 

The large-scale epidemiological studies conducted in different countries of the world have demonstrated growing number of diseases of the urinary system in children of different age groups (4, 5). The incidence of kidney pathology increases by 6-10 times in the group of children born to mothers with complicated pregnancy (6, 7).

The performed literature analysis revealed sporadic and outdated research on the influence of preeclampsia in the mother on the urinary system of the fetus and newborn, which in most cases were clinically relevant. These facts dictate the need for complex morphological studies using modern techniques in order to study the effect of various degrees of severity of preeclampsia in the mother on the urinary system of the fetus and newborn. The purpose of this work was to reveal macroscopic features of the kidneys of fetuses and newborns of the mothers whose pregnancy was complicated by preeclampsia

## Materials and methods

The macroscopic postmortem observation study involved the kidneys of 106 full-term fetuses and newborns, obtained during the autopsies at Kharkiv City Perinatal Center carried out by the employees of the Department of Pathological Anatomy of Kharkiv National Medical University. During the autopsies, the shape, texture, surface, color on the cut were assessed in each kidney, and then its weight, length, width and thickness were measured. 

The material was divided into four groups: group 1) fetuses (n=13) and newborns (n=15) of mothers with physiological pregnancy; group 2) fetuses (n=12) and newborns (n=13) of mothers whose pregnancy was complicated by mild preeclampsia; group 3) fetuses (n=13) and newborns (n=14) of mothers whose pregnancy was complicated by moderate preeclampsia; group 4) fetuses (n=13) and newborns (n=13) of mothers whose pregnancy was complicated by severe preeclampsia. The fetuses of groups 1-4 died antenatally or intranatally as a result of birth injury or severe disorders of utero-placental, umbilical blood flow, and infants-as a result of hypoxic-ischemic injury of the central nervous system. 

The diagnosis of preeclampsia was made in agreement with the orders of the Ministry of Health of Ukraine No. 620 dated 29.12.2003 «About the organization of provision of inpatient obstetric and gynecological, neonatal care in Ukraine» and No. 676 dated 31.12.2004 «About the approval of clinical protocols for obstetric and gynecological care» (8).

**Ethical consideration**

This study was approved by the commission on ethics and bioethics of Kharkiv National Medical University (4/10/03/2017). Oral consent was obtained from the parents whose children died to participate in this research.

**Statistical analysis**

Mean values in the groups were compared using a nonparametric Mann-Whitney U-test. The significance of differences between the values was taken at significance level of p<0.05. Data were analyzed using STATISTICA (data analysis software system), version 6.0, StatSoft Inc. 

## Results

**Figure 1:**
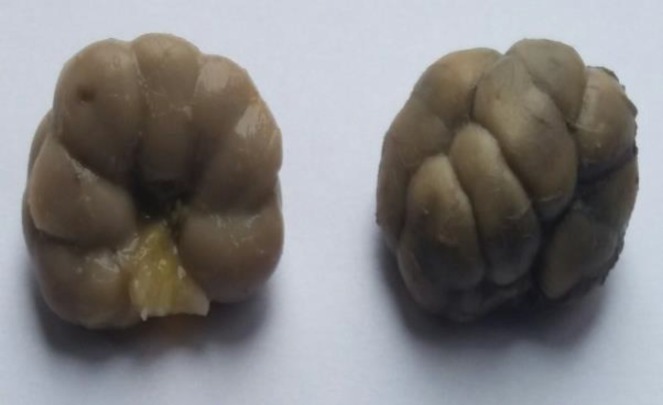
Newborn kidneys of mother whose pregnancy was complicated by severe preeclampsia

During the macroscopic study, the kidneys from the fetuses and newborns of all groups were rounded, with densely-elastic texture. The fatty kidney capsule was poorly pronounced, the thin fibrous capsule was removed without difficulty. The relief of the renal surface was distinct lobular. In fetuses and newborns from groups 1-4, the lobular surface was equally pronounced in the right and left kidneys (Figure 1). 

The results of measuring the number of lobes in kidneys of fetuses and newborns in groups are presented in table I. The mean numbers of lobes in kidneys of fetuses and newborns were significantly (p<0.05) bigger in group 4 compared with group 1. In groups 2 and 3 compared with group 1 the mean numbers of lobes in kidneys of fetuses and newborns did not differ significantly (p>0.05). In groups 1-3 the lobular surface was significantly (p<0.05) less pronounced in newborns compared with fetuses. In group 4 differences in the grade of lobular relief of the kidneys in fetuses and newborns were not identified (p>0.05). Comparison of groups 2-4 identified significantly (p<0.05) excess embryonic lobulation of the kidneys in group 4.

The cut of the kidneys demonstrated grayish cortex and reddish-bluish color of the medulla, in groups 1-3 the boundary between the layers was clearer in newborns compared with fetuses, and in group 4 the boundary between the layers was unclear not only in fetuses but also in newborns. The results of measuring the mass, length, width and thickness of the right and left kidney in fetuses and newborns are presented in table II. The analysis of the obtained values demonstrated that in groups 1 and 2 the weight of the left kidney in fetuses and newborn was significantly (p<0.05) higher compared to the mass of the right kidney; in groups 3 and 4 significant (p>0.05) differences were absent.

The analysis of the parameters of the length, width of newborns kidneys in groups 1-4 and fetal kidneys in groups 2-4 failed to reveal significant (p>0.05) difference between the values of the right and left kidneys. However, in fetuses of group 1 the length of the left kidney was significantly (p<0.05) greater than the right, and the width of the right kidney was significantly (p<0.05) greater than the left. In fetuses and newborns of all groups, the thickness of the right and left kidneys did not differ significantly (p>0.05). 

In groups 3 and 4 a significant (p<0.05) reduction of the mass, length, width and thickness of both kidneys in fetuses and newborns was found as compared with group 1. In group 2 the weight, length, width and thickness of the kidneys in fetuses and newborns did not differ significantly (p>0.05) from those in fetuses and newborns of group 1. In groups 1-3 the weight, length, width and thickness of both kidneys were significantly (p<0.05) greater in newborns compared to fetuses. In group 4 no significant (p>0.05) differences were found in the weight, length, width and thickness of the kidneys in fetuses and newborns.

**Table I:** The mean number of lobes in kidneys of fetuses and newborns

Group number	Fetus	Newborn
**1**	**10.85 ± 0.39**	**7.13 ± 0.34**
**2**	**9.92 ± 0.61**	**7.31 ± 0.32**
**3**	**10.23 ± 0.36**	**7.86 ± 0.49**
**4**	**12.15 ± 0.30**	**12.38 ± 0.42**

Data presented as mean±SD.

**Table II:** The mean value of the right and left kidney mass, length, width, and thickness in fetuses and newborns

Group number		Fetus		Newborn	
		Right kidney	Left kidney	Right kidney	Left kidney
**Mass (gr)**					
	**1**	**10.43 ± 0.24**	**11.69 ± 0.36**	**15.16 ± 0.28**	**16.10 ± 0.19**
	**2**	**10.11 ± 0.33**	**11.45 ± 0.47**	**15.02 ± 0.29**	**16.19 ± 0.29**
	**3**	**9.05 ± 0.21**	**9.18 ± 0.16**	**11.32 ± 0.28**	**11.27 ± 0.25**
	**4**	**7.92 ± 0.28**	**7.77 ± 0.24**	**8.15 ± 0.30**	**8.07 ± 0.46**
**Length (cm)**					
	**1**	**3.41 ± 0.15**	**3.93 ± 0.13**	**4.23 ± 0.23**	**4.65 ± 0.24**
	**2**	**3.26 ± 0.25**	**3.72 ± 0.28**	**3.99 ± 0.10**	**4.71 ± 0.27**
	**3**	**2.43 ± 0.12**	**2.36 ± 0.12**	**3.18 ± 0.13**	**3.07 ± 0.10**
	**4**	**1.81 ± 0.06**	**1.82 ± 0.07**	**1.96 ± 0.10**	**1.92 ± 0.05**
**Width (cm)**					
	**1**	**2.12 ± 0.15**	**1.88 ± 0.14**	**2.59 ± 0.12**	**2.26 ± 0.13**
	**2**	**1.98 ± 0.12**	**1.80 ± 0.11**	**2.52 ± 0.16**	**2.20 ± 0.14**
	**3**	**1.49 ± 0.07**	**1.47 ± 0.08**	**1.96 ± 0.08**	**1.87 ± 0.11**
	**4**	**1.32 ± 0.09**	**1.29 ± 0.10**	**1.42 ± 0.09**	**1.45 ± 0.08**
**Thickness (cm)**					
	**1**	**1.66 ± 0.14**	**1.62 ± 0.15**	**2.21 ± 0.19**	**2.19 ± 0.18**
	**2**	**1.63 ± 0.17**	**1.55 ± 0.11**	**2.25 ± 0.18**	**2.15 ± 0.17**
	**3**	**1.13 ± 0.05**	**1.15 ± 0.04**	**1.52 ± 0.07**	**1.51 ± 0.08**
	**4**	**1.02 ± 0.05**	**1.03 ± 0.06**	**0.99 ± 0.04**	**1.02 ± 0.06**

Data presented as mean±SD.

## Discussion

The present study revealed that in fetuses and newborns the relief of the renal surface was distinct lobular due to the peculiarities of the embryonic development. It is known that the human kidney is formed of several segments of nephrogenic blastema later corresponding to renal lobes. Due to the distinct boundaries between them in the form of grooves, it is possible to get a clear idea of the shape of the lobes and to determine their amount (9). It was noted that the kidney in fetus has about 12 lobes. In groups 1-3 the lobular surface was less pronounced in newborns compared with fetuses, which is a variant of the norm and results from the growth of kidneys (10). The kidneys reach their anatomical maturity after the 1^st^ yr of life, when lobulation subsides (9). The normal kidney of an adult has a smooth surface (12). In group 4, differences in the grade of lobular relief of the kidneys in fetuses and newborns were not identified. Comparison of groups 2-4 identified excess embryonic lobulation of the kidneys in group 4. Thus, the conducted analysis revealed that severe maternal preeclampsia increased the degree of the embryonic lobulation of the kidneys in fetuses and newborns. Excessive lobulation of the kidneys in fetuses and newborns is combined with various malformations of this organ (12).

One of the most important criteria for evaluating the renal status during ultrasound examination or autopsy is determining its size, as it largely varies with different pathologies (13, 14). The analysis of the obtained values demonstrated that in groups 1 and 2 the weight of the left kidney in fetuses and newborn was higher compared to the mass of the right kidney, which coincides with the findings of other researchers (2); in groups 3 and 4 differences were absent. The analysis of the parameters of the length, width of newborns kidneys in groups 1-4 and fetal kidneys in groups 2-4 failed to reveal difference between the values for the right and left kidneys. But, in fetuses of group 1 the length of the left kidney was greater than the right, and the width of the right kidney was greater than the left, which was also noted in a number of scientific studies (10, 13). 

In fetuses and newborns of all groups the thickness of the right and left kidneys did not differ significantly. In groups 3 and 4, reductions of the mass, length, width and thickness of both kidneys in fetuses and newborns were found as compared with group 1 indicating inhibition of the renal growth in children in the cases of moderate maternal preeclampsia and especially severe preeclampsia. In the cases of mild maternal preeclampsia the weight, length, width and thickness of the kidneys in fetuses and newborns did not differ from those in fetuses and newborns of group 1.

In normal pregnancy and pregnancy complicated by mild and moderate preeclampsia the weight, length, width and thickness of both kidneys were greater in newborns compared to fetuses, which is due to the growth of the organ. However, in the cases when the mother’s pregnancy was complicated by severe preeclampsia, the fetuses and newborns did not differ in the weight, length, width and thickness of the kidneys, which also suggests that severe preeclampsia in the mother decelerates kidney growth in their children. A reduction of the macroscopic parameters of the kidneys of the fetuses and newborns has been noted by numerous researchers in the case of the child development in conditions of chronic intrauterine hypoxia (15). Comparative analysis of our data and the literature data showed that kidneys in fetuses and newborns have become larger in the recent decades, which can be explained by the common trend of enlarged organs observed in today’s generation (11, 14).

Thus, our study has shown that moderate and especially severe preeclampsia of the mother delays renal growth in fetuses and newborns that manifests by a significant reduction in the weight, length, width and thickness of these organs. Mild preeclampsia in the mother does not affect the organometric characteristics of the kidneys in fetuses and newborns 
